# Irregular rhythm?

**DOI:** 10.1007/s12471-019-1231-8

**Published:** 2019-01-28

**Authors:** A. W. G. J. Oomen, F. F. J. Rozestraten

**Affiliations:** 1 Department of Cardiology, Amphia Ziekenhuis, Breda, The Netherlands; 2 Department of Cardiology, Catharina Ziekenhuis, Eindhoven, The Netherlands

## Answer

**Fig. 1:**
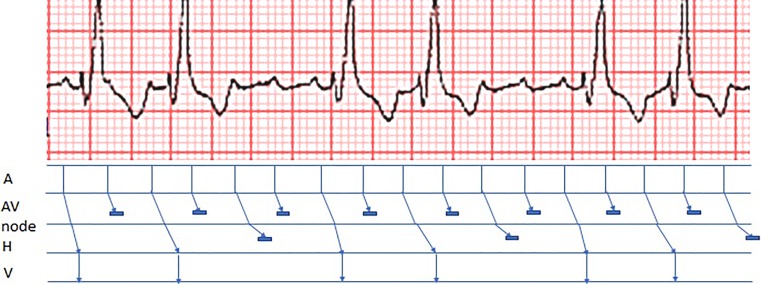
Ladder diagram showing the mechanism of varying 2:1 and 4:1 block. (*A* atrium, *AV* atrioventricular, *H* His, *V* ventricle)

The electrocardiogram recorded at presentation (Fig. 1 in the Question, 10.1007/s12471-019-1230-9) shows an atypical atrial flutter with a cycle length of approximately 300 ms. There is a remarkable variability of 2:1 to 4:1 conduction of the atrial flutter to the ventricles. This can best be explained by alternating Wenckebach periods.

Alternating Wenckebach periods are episodes of 2:1 block during which there is a gradual increase in the conduction time of conducted beats, terminating in a greater degree of block. Most episodes of alternating Wenckebach periods occur at the atrioventricular (AV) node, but some have also been reported in other structures such as the His-Purkinje system, Kent bundle and the atria. Alternating Wenckebach periods are usually attributed to block at different levels due to transverse dissociation [1]. In this case, the AV node functions physiologically with two levels of sequential block. At the first level of the AV node, one out of every two atrial impulses is blocked. At the second level, the conduction time of the conducted impulses gradually increases until impulses are blocked. This leads to a variable conduction block ratio of 2:1 or 4:1. The mechanism is depicted in Fig. 1.
